# A Review of the Development of Titanium-Based and Magnesium-Based Metallic Glasses in the Field of Biomedical Materials

**DOI:** 10.3390/ma17184587

**Published:** 2024-09-19

**Authors:** Zeyun Cai, Peng Du, Kun Li, Lina Chen, Guoqiang Xie

**Affiliations:** 1 Institute of Special Environments Physical Sciences, Harbin Institute of Technology, Shenzhen 518055, China; zexiaoyun@126.com; 2 School of Materials Science and Engineering, Harbin Institute of Technology, Shenzhen 518055, China; dupeng@szpu.edu.cn (P.D.); kun_li0514@163.com (K.L.); 3 Institute of Intelligent Manufacturing Technology, Shenzhen Polytechnic University, Shenzhen 518055, China; 4 School of Materials Science and Engineering, Shandong University of Technology, Zibo 255000, China; 5 State Key Laboratory of Advanced Welding and Joining, Harbin Institute of Technology, Harbin 150001, China

**Keywords:** metallic glasses, titanium-based, magnesium-based, biomedical materials

## Abstract

This article reviews the research and development focus of metallic glasses in the field of biomedical applications. Metallic glasses exhibit a short-range ordered and long-range disordered glassy structure at the microscopic level, devoid of structural defects such as dislocations and grain boundaries. Therefore, they possess advantages such as high strength, toughness, and corrosion resistance, combining characteristics of both metals and glasses. This novel alloy system has found applications in the field of biomedical materials due to its excellent comprehensive performance. This review discusses the applications of Ti-based bulk metallic glasses in load-bearing implants such as bone plates and screws for long-term implantation. On the other hand, Mg-based metallic glasses, owing to their degradability, are primarily used in degradable bone nails, plates, and vascular stents. However, metallic glasses as biomaterials still face certain challenges. The Young’s modulus value of Ti-based metallic glasses is higher than that of human bones, leading to stress-shielding effects. Meanwhile, Mg-based metallic glasses degrade too quickly, resulting in the premature loss of mechanical properties and the formation of numerous bubbles, which hinder tissue healing. To address these issues, we propose the following development directions: (1) Introducing porous structures into titanium-based metallic glasses is an important research direction for reducing Young’s modulus; (2) To enhance the bioactivity of implant material surfaces, the surface modification of titanium-based metallic glasses is essential. (3) Developing antibacterial coatings and incorporating antibacterial metal elements into the alloys is essential to maintain the long-term effective antibacterial properties of metallic biomaterials. (4) Corrosion resistance must be further improved through the preparation of composite materials, while ensuring biocompatibility and safety, to achieve controllable degradation rates and degradation modes.

## 1. Introduction

Human society’s development has propelled advances in materials, and since the beginning of the 21st century, the increasing demand for high-strength metal materials has become more urgent with rising performance requirements.

Biomedical metallic materials are widely used for supporting or replacing structural organs in the human body due to their high yield strength, ductility, fatigue strength, and fracture toughness. These materials are employed for protecting and repairing human tissues, as well as in artificial organs and orthopedic medical devices. Biomedical metallic materials are primarily used for the protection and repair of bones, joints, teeth, vascular stents, and bridges.

Currently, biomedical metallic materials can be classified into two categories:

(1) Non-degradable biomedical metallic materials: Non-degradable biomedical metallic materials are represented by titanium and titanium alloys, stainless steel, and cobalt-based alloys.
(2) Degradable biomedical metallic materials: Degradable biomedical metallic materials are represented by magnesium and magnesium alloys, iron-based alloys, and zinc alloys.

**Figure 1:**
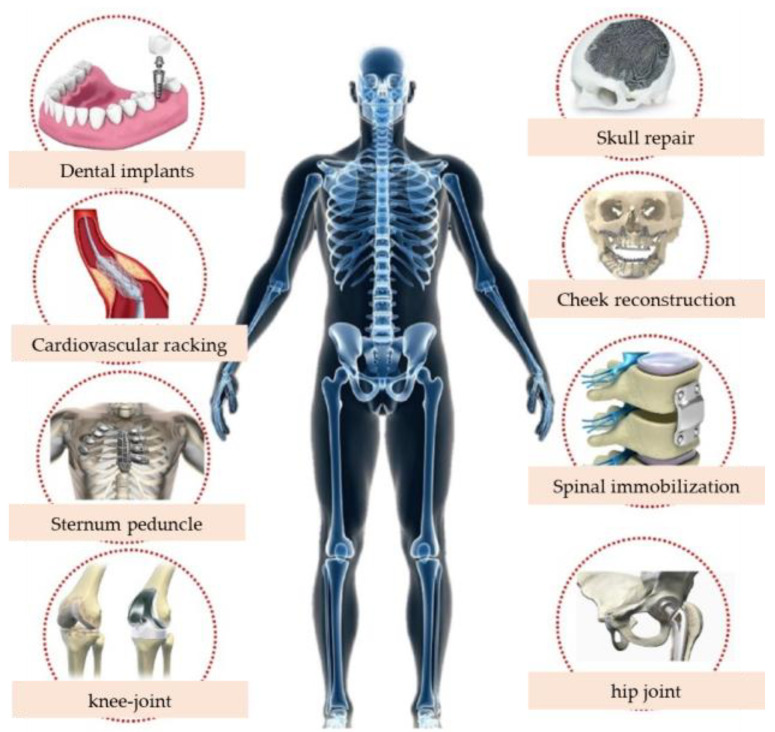
Application examples of biomedical metal materials.

In addition to mechanical properties, chemical and physical characteristics, concepts such as biocompatibility, stress shielding, bioactivity, and bone induction, are considered crucial factors in the design of medical implants. Biomedical implant materials need to meet various requirements, including excellent corrosion resistance in the body environment, a favorable combination of high strength and low Young’s modulus, excellent wear resistance and fatigue resistance, good ductility, and non-cytotoxicity. They must also withstand cyclic biomechanical stress during their service life and have the potential to support bone regeneration. Among these requirements, the most critical is the biocompatibility of the alloy, followed by its mechanical properties. Figure 1 shows examples of biomedical metal materials used in the human body. As long-term implant materials, metals have been widely used in dental implants, cardiovascular stents, the sternum, knee joints, cranial repairs, facial reconstruction, spinal fixation, and hip joints, among others.

### 1.1. Biocompatibility

Biocompatibility is defined as the ability of a biomaterial, prosthesis, or medical device to perform with an appropriate host response under specific application conditions. Examples of appropriate host responses include being highly non-toxic, non-inflammatory, and non-allergenic, having antibacterial properties, and facilitating normal and uncomplicated healing [1]. When an implant material is introduced into the human body, interactions occur between the implant and the host which determine the body’s tolerance to the implant. The primary response to any foreign implant entering the body is known as the rejection reaction [2]. Factors affecting rejection reactions include toxicology, mechanical compatibility, the proliferation of biological entities on the surface of biomaterials, and interactions between cells and biomaterials [3]. From a toxicological perspective, implant materials should not release any substances into the human body unless intentionally designed to do so [4]. Therefore, implant materials must be chemically stable within the human body. However, the release of metal ions and other constituents into tissues or body fluids is inevitable, even if the implant material is considered to have excellent corrosion resistance. This is because implant materials are usually exposed to rejection, corrosive, and protein-rich environments [5]. Wear-induced metal ion release can trigger various adverse biological responses, such as tissue allergies, localized blood vessel damage-supplying bones, osteonecrosis, and implant loosening [6]. Leaching chemicals from implants may inhibit cell proliferation, leading to undesirable implant outcomes. Incompatible metal ion deposits around the implant site may also cause tissue allergies and toxicity, leading to the inflammation and death of surrounding tissues [7]. Therefore, it is crucial to develop implant materials free of cytotoxic elements, especially for patients with metal allergies. At the initial stage of developing biomedical metallic materials, it is essential to focus on whether the alloying elements may cause adverse effects, such as carcinogenicity, mutagenicity, genotoxicity (DNA damage), cytotoxicity (cell damage/death), allergic reactions, and whether they can withstand corrosive biological environments. While a single metal cannot determine the final alloy’s biocompatibility, reasonable predictions can be made. Based on previous research and clinical application analysis, elements such as Ti, Au, Sn, Ta, Nb, Ru, and Zr are considered to have high biocompatibility [8]. Hf and Re show potential but require further rigorous investigation. Other reviewed elements are considered unsatisfactory.

### 1.2. Mechanical Properties

In addition to the biocompatibility of alloying elements, it is also essential to consider the mechanical environment in which the alloy will be used. For the human body, implant materials will serve long-term in a warm, corrosive environment and be in close contact with soft tissues. Therefore, the mechanical biocompatibility of the implant must be considered, with the most crucial factor being Young’s modulus [9]. From the perspective of mechanical biocompatibility, important parameters such as Young’s modulus, tensile strength, and fatigue strength not only determine the ability of the implant material to provide its intended function but also its mechanical biocompatibility, which is critical to the service life of implants once inside the human body. In soft tissues, mechanical biocompatibility is less of a concern, as soft tissues are non-load-bearing and can adapt to implants. For example, cardiovascular stents rely on a high Young’s modulus to provide the necessary anti-collapse capability [10]. However, for implants used to replace or repair load-bearing bones, such as hip and knee joint implants, mechanical biocompatibility is of significant concern. If the implant’s Young’s modulus is higher than that of the load-bearing bone, the force cannot be effectively transferred to the bone tissue, leading to bone resorption and loosening around the implant, which eventually causes the implant to loosen or fracture, resulting in implant failure. This phenomenon is known as the stress-shielding effect [11]. Different bone tissues in various parts of the human body have distinct requirements for implant materials, as shown in Table 1. When designing and fabricating implant materials, focusing solely on one requirement would be biased; it is necessary to match the implant material with the specific implantation site, including matching the modulus and mechanical properties.

### 1.3. Other Requirements

Biomedical metallic materials need to possess high corrosion and wear resistance to prevent the release of incompatible metal ions that could trigger allergic reactions and toxicity when implanted in the human body [13]. High corrosion and wear resistance can also enhance the material’s longevity. Additionally, these materials should have good osseointegration capabilities, which means forming a direct interface with bone tissue without interfering with soft tissue. It is worth noting that surface chemistry, surface roughness, and surface morphology play a crucial role in achieving good osseointegration [1].

### 1.4. Metallic Glass

**Figure 2:**
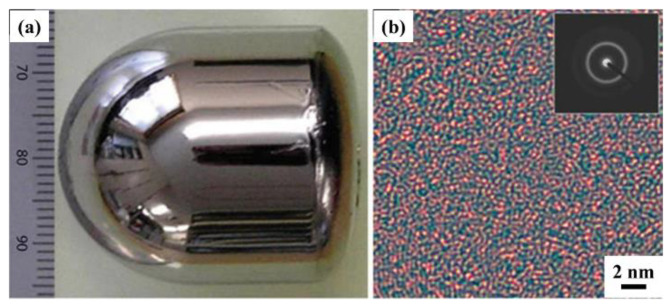
Typical morphology and microstructure of cast BMG (**a**) Typical morphology and (**b**) corresponding HRTEM image and SAED pattern [20].

As a representative of new high-strength structural materials, bulk metallic glasses (BMGs) [14] have demonstrated unique advantages in engineering fields. The microstructure of crystalline alloys exhibits regular periodic arrangements in three-dimensional space [15,16]. In contrast, metallic glasses are formed through the process of rapid cooling of molten alloys, which suppresses the nucleation and growth of crystals, thereby maintaining the disordered structure of the molten metal to form metallic glass. This rapid cooling prevents the rearrangement of atoms within the molten metal, resulting in metallic glasses lacking the periodic symmetry found in crystalline alloys. Metallic glasses are specifically disordered non-crystalline alloys (amorphous alloys) [17] and do not possess structural defects such as the dislocations or grain boundaries commonly found in crystalline alloys [18]. Because metallic glasses are composed of metallic elements and exhibit a glassy microstructure, they combine the advantages of metals and glasses [19]. The typical morphology of as-cast metallic glasses and corresponding HRTEM images and SAED patterns are shown in Figure 2. The typical as-cast metallic glass morphology, along with their corresponding HRTEM images and SAED patterns, are shown in Figure 1. Figure 2a displays the appearance of the as-cast metallic glass sample, which exhibits a metallic luster; Figure 2b shows the microstructure of the metallic glass, where various atoms are uniformly distributed with no noticeable aggregation.

**Figure 3:**
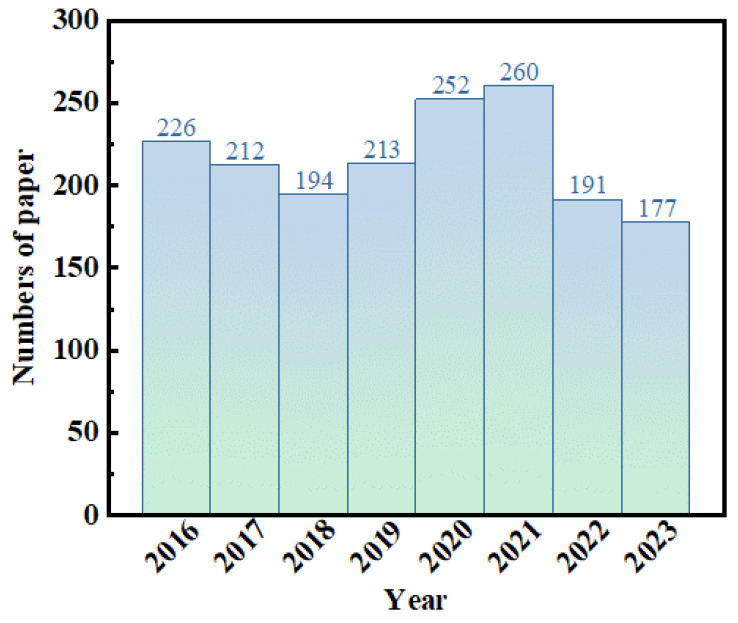
Graph of the number of BMG research papers versus the year.

Metallic glasses lack an ordered microstructure, leading to a higher Gibbs free energy compared to equilibrium crystalline alloys. Therefore, metallic glasses exhibit excellent properties such as high strength, high hardness, high elastic limit, high fracture toughness, good corrosion resistance, and fatigue resistance [21]. Researchers in material science and medicine have conducted extensive research on metallic glasses in the field of medical instruments [22]. Their excellent physical properties and corrosion resistance make metallic glasses promising biomaterials with development potential. Currently, implant materials applicable in the biomedical field include non-degradable Ti-based [23,24] and Zr-based [25,26] metallic glasses, as well as degradable Mg-based [27], Fe-based [28], and Ca-based [15] metallic glasses. We counted the number of papers studying BMGs (bulk metallic glasses) from 2016 to 2023, as shown in Figure 3. It can be seen from this Figure that researchers’ interest in BMGs has remained consistently high.

## 2. Preparation and Application of Bulk Metallic Glasses in Biomedical Applications

Metal materials possess high strength, toughness, excellent fatigue resistance, and ease of processing and forming, making them the most widely used and extensively applied type of biomedical material in clinical settings. They are primarily used for hard tissue repair and replacement, such as artificial bones, joints, dental roots, and bone fixation devices, as well as vascular or esophageal stents for cardiovascular and soft tissue repair. Implantable materials can be classified into long-term implants and degradable implants. Currently, long-term implant materials used in clinical applications mainly include 316 L austenitic stainless steel, Co-Cr alloys, and titanium alloys (commercially pure titanium and Ti-6Al-4V), while degradable implant materials mainly include magnesium alloys, zinc alloys, calcium alloys, and others. Long-term implant materials are often used as bone substitutes and remain inside the body permanently or for a long period to provide structural support and bear loads. Therefore, besides having high strength, these materials also need to exhibit excellent corrosion resistance and wear resistance. To avoid the “stress-shielding” effect, they should also have a lower Young’s modulus. However, 316 L austenitic stainless steel (210 GPa) and Co-Cr alloys (240 GPa) both have high Young’s moduli and exhibit poor biocompatibility and corrosion resistance. The metal ions released from their degradation in the body can easily cause inflammatory reactions. In contrast, titanium alloys have excellent corrosion resistance, biocompatibility, and mechanical properties, making them the mainstream choice in clinical applications and product development.

Metallic glass, with no point defects, dislocations, or grain boundaries, has superior mechanical properties such as ultra-high strength, low Young’s modulus, high hardness, and excellent corrosion and wear resistance. Titanium-based metallic glass, which is free from biologically toxic and harmful elements such as Be, Ni, and V, not only possesses outstanding mechanical properties but also has a low density and excellent biocompatibility, making it highly promising for biomedical applications. Long-term implantable titanium-based metallic glass must meet the following conditions: (1) It should not contain elements like Be, Ni, V, or Al, which are toxic and harmful to the human body, ensuring excellent cellular safety and biocompatibility. (2) The strength and Young’s modulus of the material should match those of human bone. (3) It should have a strong glass-forming ability or suitable preparation methods to overcome size limitations. These can be achieved by developing metallic glass systems that are free of toxic and harmful elements, have a lower Young’s modulus, and possess a strong glass-forming ability. Additionally, the Young’s modulus can be reduced by introducing a porous structure within the material, and the size limitations can be addressed through appropriate preparation techniques.

**Figure 4:**
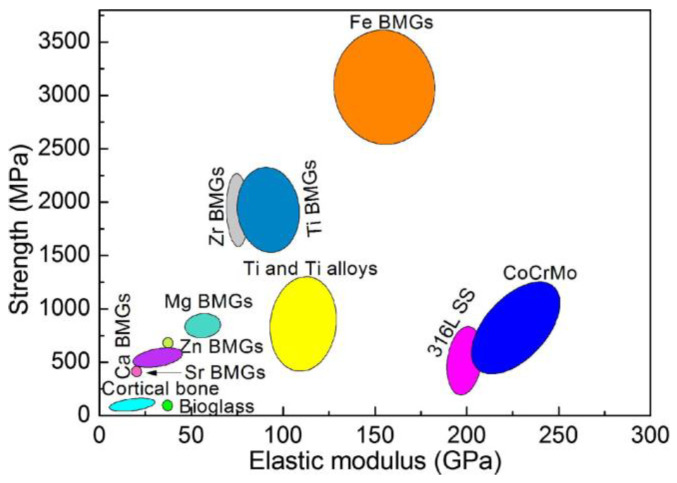
Mechanical comparisons of conventional bioglasses, biometallic alloys, and the novel developed biomedical BMGs [29].

To date, the development and research on amorphous alloys have a history of more than eighty years. Records of amorphous alloys first appeared in 1937, when German scholar Krammer developed thin strips of this special alloy using chemical vapor deposition. The breakthrough with bulk amorphous alloys occurred in 1962, when American scholars Duwez et al. successfully prepared Au–Si amorphous alloys using the melt-quenching method, with cooling rates of 10^5^ K/s to 10^6^ K/s. It was found in their research that amorphous alloys significantly improved mechanical properties compared to crystalline alloys with the same composition. Figure 4 illustrates the compression strengths and Young’s modulus for conventional bioglasses, biometallic alloys and the novel developed biomedical BMGs. By comparing the compressive strength and Young’s modulus of metallic glasses based on iron, magnesium, and titanium with those of stainless steel, Co-based alloys, and titanium alloys, it is evident that metallic glasses have higher compressive strength and lower Young’s modulus. Therefore, the comparison of compressive strength and Young’s modulus indicates that metallic glasses are more suitable as biomedical implant materials than conventional alloys. In contrast, biomedical metal glass has the comprehensive characteristics of biological glass and biometal, and the characteristics of high strength and low Young’s modulus make this type of material have a good prospect as a medical metal.

**Figure 5:**
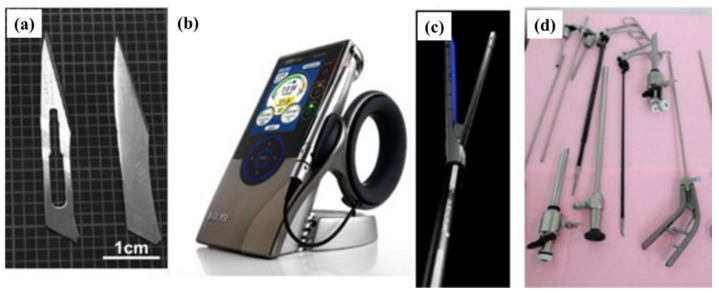
Illustrations of biomedical implants and devices made out of BMGs [29]. (**a**) A commercial martensitic steel surgical blade coated with ZrCuAlAgSi metallic glass (left) and a ZrCuAlAgSi metallic glass surgical blade (right); (**b**) a diode dental laser system using liquid metal in its casing; (**c**) a metallic glass medical suture anvil; (**d**) liquid metal alloys in minimally invasive medical devices.

Researchers have proposed many preparation strategies for amorphous alloys, all agreeing that sufficiently rapid cooling rates are necessary. Under the conditions at that time, it was very difficult to mass-produce large-sized bulk amorphous alloys, and the amorphous alloys that could be prepared were generally thin strips smaller than 1 mm, limiting their practicality [20]. The research group led by A. Inoue [30] from Japan made significant advancements in the development of large-sized amorphous alloys, introducing the concept of bulk metallic glasses. They successfully developed relatively large-sized Mg-based and Fe-based metallic glasses in 1988 and 1995, respectively. Subsequently, with copper mold casting as the primary method, further developments were made in the development of bulk metallic glasses. The Pd-based metallic glass system developed by Chen et al. only requires cooling rates of about 10 K/s, highlighting significant differences in cooling rate requirements among different metallic glass systems. As research deepened, several empirical principles for designing and developing metallic glasses were proposed, with the most widely recognized being the “three principles” by A. Inoue [31]: (1) the number of elements in the alloy should be three or more; (2) the atomic radii within the alloy should differ by at least 12%; (3) the mixing enthalpy between the elements should be negative. Guided by these principles, metallic glass systems with critical diameters reaching 2 cm for Zr-, Zr-Be-, Mg-, La-, Ni-, and Cu-based metallic glasses and critical diameters of 1 cm for Fe-based and Ti-based metallic glasses have been developed [30]. Besides the three principles by A. Inoue, the deep eutectic principle is also widely accepted among researchers. This principle suggests that the ability of an alloy system to observe deep eutectic points is a crucial criterion for determining whether the system can form metallic glasses, with metallic glasses more likely to form in regions near deep eutectic points. With the development of processes such as copper mold suction casting, mold pressing casting, and directional solidification, the development of bulk metallic glasses has seen new advancements and has gradually emerged in practical applications in many fields. Figure 5 depicts the recent applications of metallic glasses in the biomedical field. As research progresses, more metallic glasses may move toward clinical applications in the future. Figure 5a shows a commercial martensitic steel surgical blade coated with a ZrCuAlAgSi metallic glass coating (left) and a ZrCuAlAgSi metallic glass surgical blade (right); Figure 5b depicts a diode dental laser system using liquid metal in its casing; Figure 5c shows a metallic glass medical suture anvil; and Figure 5d displays liquid metal alloys used in minimally invasive medical devices. Compared to conventional alloys, metallic glasses have a higher hardness, making the blades and instruments more stable during surgery.

Since the discovery of the first metallic glass system, this new alloy system has quickly gained recognition from researchers in various industries due to its excellent comprehensive properties, such as high strength, high hardness, high elastic limit, high fracture toughness, good corrosion resistance, and fatigue resistance [32]. It has achieved a breakthrough in size, progressing from micron-sized ribbons or wires to millimeter-sized bulk forms. The physical properties of metallic glasses vary across different systems, leading to significant differences in their application fields. Ti-based bulk metallic glasses are mainly used in load-bearing parts such as bone plates and screws for long-term implants [31]. Zr-based bulk metallic glasses, with their excellent glass-forming ability and outstanding mechanical properties, show great potential in areas like artificial joints and femoral head supports. Mg-based metallic glasses, due to their mechanical properties that match human tissues and their degradability, are primarily used in degradable bone screws, bone plates, and vascular stents.

## 3. Research Progress of Titanium-Based Metallic Glasses

Titanium-based bulk metallic glasses (Ti-BMGs) have shown superior performance compared to their crystalline alloy counterparts in many aspects, making them promising candidates for long-term human implant materials in the future. The advantages of using Ti-BMGs as orthopedic implant materials can be summarized as follows [31,33]: (1) High strength and hardness: medical implant materials need to possess a good load-bearing capacity and high wear resistance. (2) Low Young’s modulus: this ensures better load transfer from the implant to the surrounding bone, reducing the harm caused by “stress shielding”. (3) Excellent corrosion resistance: this lowers the release rate of metal ions during service in the human body. (4) Superior formability: due to their unique superplasticity in the supercooled liquid region, metallic glasses can be shaped according to actual needs, which is significant for the processing of biomaterials.

Although methods for preparing metallic glasses are continuously increasing, rapid cooling methods, especially copper mold casting, still play the most crucial role. Due to the current limitations in cooling rates, not all alloy systems can form metallic glasses. Experimental results have shown that while most bulk metallic glasses are multi-component alloys, all developed metallic glasses can be traced back to binary alloys. Research has found that binary systems, Ti-X (X = Cu, Be, Pd, Si, Co, Ni), which can form deep eutectics with Ti, are the starting framework for all multi-component bulk metallic glasses. Based on the binary alloy systems and A. Inoue’s three principles, increasing the complexity of the alloy system by substituting elements with similar properties can further develop Ti-BMGs with an excellent glass-forming ability [31].

Although Ti-BMG systems are becoming increasingly diverse, many elements that facilitate metallic glass formation do not necessarily possess biocompatibility, which limits the design of biocompatible Ti-BMGs. For example, elements like Ni, Be, and Al, which are commonly found in Ti-BMGs, are highly allergenic or toxic to the human body and may even be carcinogenic. However, once these elements that enhance metallic glass formation are excluded, the reported Ti-BMGs are quite limited and can be mainly categorized into two types.

### 3.1. Ti-Zr-Cu-Pd System Metallic Glass

**Figure 6:**
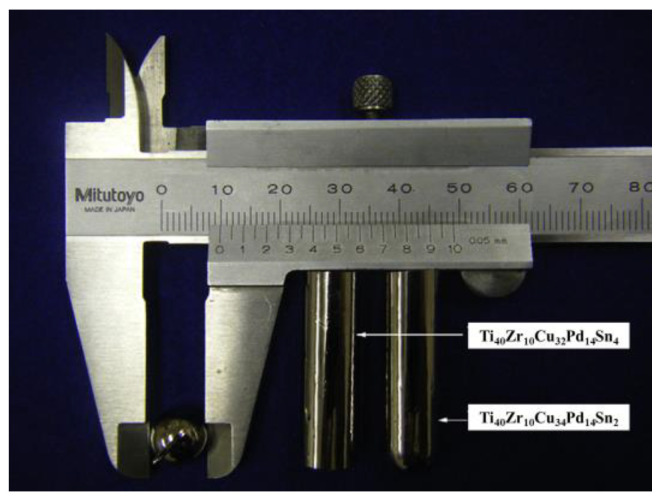
Outer surface of cast Ti_40_Zr_10_Cu_34_Pd_14_Sn_2_ and Ti_40_Zr_10_Cu_32_Pd_14_Sn_4_ bulk glassy alloy rods [35].

The Ti-Zr-Cu-Pd system represents a significant breakthrough in the development of Ti-BMGs that do not contain toxic elements. This system began with the Ti-Zr-Cu-Pd BMG formed in 2007 [34]. The basic framework of this alloy system is the Ti-Cu binary alloy. Since Pd has similar properties to Cu, it is introduced to partially replace Cu to improve its glass-forming ability (GFA). Based on this design concept, a series of Ti-Zr-Cu-Pd BMGs with critical diameters up to 6 mm have been developed, among which the Ti_40_Zr_10_Cu_36_Pd_14_ BMG has a maximum critical diameter of 7 mm. To further enhance the GFA, Zhu et al. [35] conducted a series of studies on the effect of adding Sn to the alloy. After adding 2–4 at.% Sn, the critical diameter of the metallic glass significantly increased to 10 mm, which is larger than the critical diameter of most Ti-BMGs that do not contain Be. Figure 6 shows the appearance of cast Ti_40_Zr_10_Cu_34_Pd_14_Sn_2_ BMG and Ti_40_Zr_10_Cu_32_Pd_14_Sn_4_ BMG rods, with the diameter of the Ti_40_Zr_10_Cu_34_Pd_14_Sn_2_ BMG rod exceeding 12 mm. Both the Cu mold casting and powder metallurgy methods have demonstrated the excellent performance of Ti_40_Zr_10_Cu_32_Pd_14_Sn_4_ BMG, with a compressive strength of up to 2060 MPa [36,37]. In addition to Sn, elements such as Si [38], Nb [39], and Co [40] have also been successively added to the Ti-Zr-Cu-Pd alloy. The reported critical diameters for Ti-Zr-Cu-Pd-Si BMG, Ti-Zr-Cu-Pd-Nb BMG, and Ti-Zr-Cu-Pd-Co BMG are 5 mm, 2 mm, and 10 mm, respectively.

Furthermore, Ti-Zr-Cu-Pd-system metallic glasses exhibit excellent corrosion resistance [24,37]. Whether through casting or powder metallurgy, this series of metallic glasses shows a wide passivation region and high pitting potential in electrochemical tests. Compared to commercial titanium alloys, they have higher corrosion potentials and lower corrosion currents. Even with the introduction of a porous structure using NaCl as a pore-forming agent, this system of metallic glasses still demonstrates strong corrosion resistance, highlighting significant application potential [41]. In parallel, there have been numerous studies on the biocompatibility of Ti-Zr-Cu-Pd BMGs. These materials have shown good biocompatibility in both in vivo and in vitro experiments. Oak et al. [42] conducted a series of studies on the corrosion behavior and biocompatibility of Ti_45_Zr_10_Cu_31_Pd_10_Sn_4_ BMG, showing that this metallic glass exhibited good biocompatibility in osteoblast culture tests. Similarly, in the work of Alethea Liens et al. [43], the cell viability of osteoblasts (MG63) and fibroblasts (HDFa) on Ti_40_Zr_10_Cu_36_Pd_14_ BMG and a Ti-6Al-4V alloy were comparable, and the growth stages of the osteoblasts on both alloy surfaces were also very similar. Xie et al. [44] evaluated the in vivo biocompatibility of Ti_40_Zr_10_Cu_34_Pd_14_Sn_2_ BMG rods by implanting them subcutaneously in the backs and into the femoral condyles of rats. There was no significant inflammatory or foreign body reaction observed around the implanted titanium-based metallic glass rods. No congestion or edema was found in the subcutaneous or bone tissues. Subcutaneous samples showed a mild fibrous capsule reaction with no inflammatory cell infiltration. Toluidine blue-stained sections of bone samples indicated that new bone could regenerate directly on the metallic glass surface, covering the entire surrounding area. The titanium-based metallic glass rods demonstrated good biocompatibility in both soft and hard tissues and showed excellent osteoconductivity when implanted in bone tissue [44,45].

The excellent comprehensive properties of Ti-Zr-Cu-Pd-system metallic glasses make them promising candidates for biomedical materials. However, their higher Young’s modulus compared to the human bone may lead to stress-shielding effects, and this is currently the biggest obstacle to their application. Additionally, the presence of the precious metal Pd in the alloy system results in high material costs, making it unsuitable for short-term implants such as internal fixation devices for fractures.

### 3.2. Ti-Si System Metallic Glass

**Figure 7:**
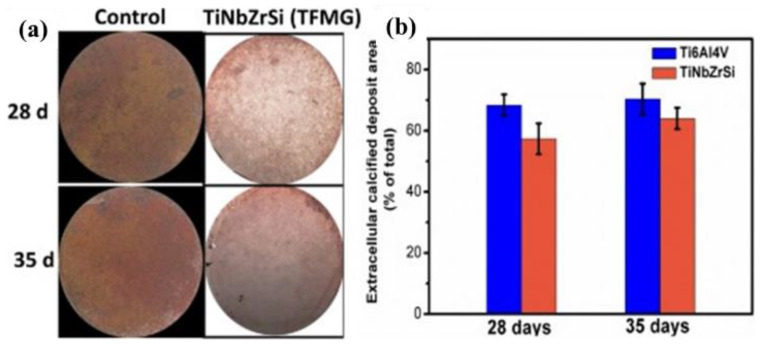
Calcified deposits by MC3T3-E1 cells on Ti-6Al-4V and Ti-Nb-Zr-Si thin-film metallic glass [53] (**a**) after alizarin red S staining; (**b**) proportion of calcified area on Ti6AlV and TiNbZrSi TFMG to total area of specimens.

Si is an ideal element for enhancing the glass-forming ability (GFA) of Ti-based metallic glasses, whether based on deep eutectic theory or A. Inoue’s three principles. Moreover, Si exhibits many biological functions compared to other added elements. Si has a good stimulating effect on bone formation and is widely considered an essential nutrient for healthy bone metabolism, including the regulation of bone mineralization. Ti-Si alloys are not only regarded as successful replacements for tissues and damaged organs but are also used as excellent surface coatings for implants. Studies have shown that Ti-Si coatings can enhance the early bonding between titanium alloys and human bone. Given these advantages, the more cost-effective Ti-Si-system metallic glasses have garnered attention. Oak et al. [46,47] successively prepared two different compositions of amorphous ribbons: Ti_45_Zr_10_Pd_40_Si_5_ and Ti_60_Zr_10_Ta_15_Si_15_. The former has a tensile strength and dynamic Young’s modulus of 2390 MPa and 88 GPa, respectively, while the latter exhibits a Vickers hardness and Young’s modulus of 745 HV and 73 GPa. Both alloy ribbons also demonstrated superior corrosion resistance compared to CP-Ti and Ti-6Al-4V. To address issues with Oak et al.’s Ti-Zr-Si-Ta amorphous alloy, such as high liquidus temperature (Tl > 1823 K), narrow supercooled liquid region (SCL < 50 K), and a weak GFA, H.C. Lin et al. [48] conducted further research. Among the series of Ti-Zr-Si-Ta alloys prepared, the Ti_42_Zr_40_Ta_3_Si_15_ alloy had the best overall performance. This alloy exhibited excellent toughness in bending tests, showing no fractures even after bending beyond 180 degrees. Additionally, this amorphous alloy has a glass-transition temperature of 799 K, a crystallization temperature of 898 K, and a supercooled liquid region of up to 99 K. It maintained good thermal stability even after isothermal annealing at 823 K for over 3000 s. In NaCl solution, the corrosion current density of this amorphous alloy was much lower than that of 304 stainless steels. Guided by A. Inoue’s three principles, Bai et al. [49] used single-roller melt-spinning technology to prepare Ti_70_Zr_6_Fe_7_Si_17_ and Ti_64_Zr_5_Fe_6_Si_17_Mo_6_Nb_2_ amorphous alloy ribbons. Both alloys primarily consist of an amorphous phase, with nanocrystals mainly composed of β-Ti and Ti_5_Si_3_ dispersed within the amorphous matrix. The addition of Mo and Nb improved the glass-forming ability. Notably, after being cultured in simulated body fluid for 15 days, both alloys had dense apatite layers deposited on their surfaces. The atomic ratio of n(Ca)/n(P) was about 1.6/1, indicating good bioactivity, which enabled the amorphous alloys to form a biological fixation with human bone tissue, a feature unattainable by most inert titanium alloys. M. Calin et al. conducted a systematic analysis of the biosafety and glass-forming trends of 27 elements. Based on biosafety and glass-forming favorability, Ti_75_Zr_10_Si_15_ and Ti_60_Nb_15_Zr_10_Si_15_ were selected for further study. Only the Ti_60_Nb_15_Zr_10_Si_15_ quaternary alloy could achieve a fully amorphous ribbon when highly superheated, whereas the Ti_75_Zr_10_Si_15_ ternary alloy contained BCC-phase nanocrystals in addition to the amorphous matrix. Ti-(Nb)-Zr-Si metallic glasses and nanocomposites, without adding toxic elements, demonstrated excellent properties, including high hardness and strength (>2000 MPa), high specific strength, and excellent corrosion resistance. Both alloys had passivation current densities below 3 × 10^−7^ A/cm^2^, an order of magnitude lower than CP-Ti. Further studies showed that after alkali–heat treatment, the developed metallic glass ribbons had a hydroxyapatite-inducing ability comparable to CP-Ti. Although the addition of Nb slightly reduced their bioactivity, it improved the alloys’ corrosion resistance. The nearly single-phase glassy alloy containing Nb exhibited similar corrosion performance to the single-phase β-type Ti-40Nb alloy [50,51]. Using magnetron sputtering, J. J. Lai et al. [52] prepared thin-film Ti-Ta-Zr-Si metallic glasses. Compared to conventional pure titanium and Ta metals, the developed metallic glass had higher hardness and a lower modulus, better matching the mechanical properties of human bone. MTS results indicated that in a 72 h in vitro test, the metallic glass displayed better cell viability and attachment rates than pure titanium and Ta surfaces. Similarly, S. Thanka Rajan et al. prepared Ti-Nb-Zr-Si metallic glass films on a Ti-6Al-4V alloy via sputtering. The study found that Ti-Nb-Zr-Si metallic glass films significantly improved the corrosion resistance and biocompatibility of the titanium alloy. The influence of the metallic glass film on MC3T3-E1 cell calcification is shown in Figure 7. Figure 7 shows that the MC3T3-E1 cells were cultured for 28 and 35 days on the specimens and the color of the control group was darker than that of TFMG. The culture after differentiation-induction on the sample shows scarlet when stained, which means the degree of calcification of the uncoated sample (control) was little higher than that of the TFMG. Figure 7b shows the ratio of the extracellular calcified deposit area of two samples. The outcome shows that the calcification ratio of the control sample was similar to that of TFMG, which demonstrates that the control and TFMG were beneficial to the calcium mineralization of MC3T3-E1 cells.

**Figure 8:**
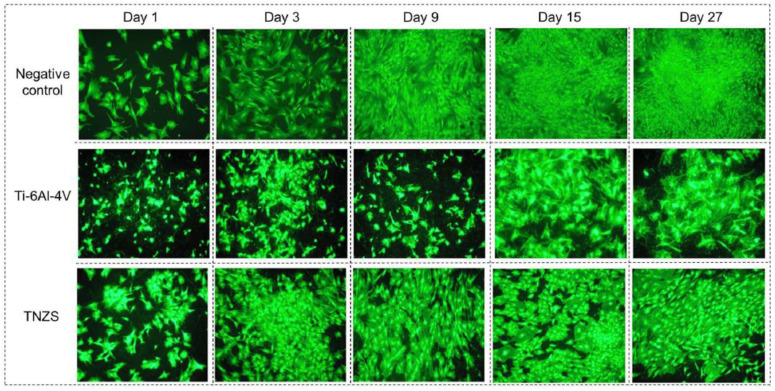
HOC proliferation and growth (×100) of negative control, Ti–6Al–4V, and TNZS at 1, 3, 9, 15, and 27 days [55].

Camelia Gabor et al. [54] prepared Ti_64_Zr_10_Si_15_Nb_11_ and Ti_56_Zr_10_Si_15_Nb_19_ titanium-based amorphous ribbons using the melt-spinning method, and the amorphous Ti_64_Zr_10_Si_15_Nb_11_ variant may exhibit better wear resistance and resistance to plastic deformation. Aydin Bordbar-Khiabani et al. [55] developed a novel Ti–Nb–Zr–Si (TNZS) alloy and compared it with commercially pure titanium and the Ti–6Al–4V alloy. They tested the alloys in different media: phosphate-buffered saline (PBS) as a normal medium, PBS/hydrogen peroxide (H_2_O_2_) as an inflammatory medium, and PBS/H_2_O_2_/albumin/lactate as a severe inflammatory medium. The results showed that the TNZS alloy exhibited superior corrosion resistance under all conditions. The developed TNZS alloy was subsequently subjected to cell culture studies to evaluate its biocompatibility. Compared to Ti–6Al–4V, it showed better cell–material interactions in vitro. Figure 8 shows HOC proliferation and growth of three samples of negative control, Ti-6Al-4V, and TNZS at 1, 3, 9, 15, and 27 days. It shows that cells on TNZS reproduced much better than that on Ti–6Al–4V alloy, but lower than that on the negative control. Therefore, compared with commercial titanium alloys, TNZS is even more biocompatible.

J.L. Ke et al. [56] prepared Ti-Zr-Si thin films using electrochemical deposition, and the corrosion resistance gradually improved with increasing Ti content. The passive current results indicated that amorphous alloys could form more protective and denser passive films on the surface of metallic glass compared to crystalline materials. Additionally, the mechanical properties of Ti-Zr-Si metallic glass thin films are superior to those of crystalline films. Compared to the titanium-based metallic glasses of other systems, the Ti-Si system has been relatively less studied. Due to the lack of late-transition elements, their actual metallic glass-forming ability is not high, and so far, these alloys are almost limited to ribbons or thin films, significantly restricting their use as structural materials. Titanium-based metallic glasses, with their unique amorphous structure, exhibit superior comprehensive properties compared to traditional titanium alloys, showing great potential for application in the biomedical field. However, there are still some issues with using titanium-based metallic glasses as biomaterials. Titanium-based bulk metallic glasses (BMGs) often contain elements that are toxic to the body (e.g., Ni, Be, Al). Moreover, their Young’s modulus does not match that of human bone, and they lack bioactivity.

### 3.3. Prospects of Titanium-Based Metallic Glass in the Biomedical Field

Compared to traditional titanium alloys, titanium-based metallic glass (Ti-BMG) has made significant breakthroughs in performance. However, it still cannot replace pure titanium and Ti-6Al-4V alloys in the medical market. Before Ti-BMGs can be fully adopted in clinical applications, several issues need to be addressed, including differences in their Young’s modulus value and antibacterial properties compared to conventional medical materials. To make Ti-BMGs more suitable as biomedical metal materials, research should focus on the following areas:

(1) Reducing Young’s Modulus: When titanium-based metallic glass is implanted into the human body, it often bears more load than bone tissue when subjected to external stress. According to Wolff’s law, human bones grow healthier only when needed. Over time, implants with a higher Young’s modulus can hinder new bone formation and even cause the surrounding natural bone to atrophy. Introducing porous structures has proven to be an effective way to reduce the Young’s modulus of metals; however, these structures can significantly degrade material properties, such as strength and corrosion resistance, which may compromise the long-term serviceability of titanium alloys. Therefore, introducing porous structures into Ti-BMGs is an important research direction for reducing Young’s modulus.
(2) Improving Biocompatibility: Most biomedical Ti-BMGs are biologically inert, meaning they cannot form biological bonds with human bone tissue, leading to poor osseointegration. This is a significant cause of the aseptic loosening of implant materials. To enhance the bioactivity of implant surfaces, various surface-modification methods, such as ion implantation, electrochemical reactions, and hydrothermal treatments, have been proposed. However, these methods often have limited effectiveness on complex-structure metallic materials. Thus, the surface treatment of Ti-BMGs to enhance biocompatibility is another key research direction.
(3) Enhancing Antibacterial Properties: Implant failures due to bacterial infections are not uncommon. Improving the antibacterial properties of medical materials is one of the current focuses in medical material development. Compared to applying antibacterial coatings, adding antibacterial metal elements to alloys can provide longer-lasting antibacterial effects and is simpler and more efficient. Due to its low toxicity, excellent cell compatibility, and low cost, copper (Cu) is the most favored antibacterial element. However, in titanium alloys, Cu only exhibits antibacterial effects when present in a specific form (*ε*-Cu phase); when dissolved in the solid solution, its antibacterial effectiveness is significantly reduced. Therefore, there is an urgent need for a solution to balance the antibacterial activity and mechanical properties of titanium alloys.

## 4. Application of Mg-Zn-Ca Metallic Glasses and Composites in the Biomedical Field

The purpose of degradable implant materials is to function during the treatment period without the need for a second surgery to remove them. Therefore, these materials must not only possess certain mechanical properties but also have a specific degradation rate, with degradation products that do not cause toxicity or inflammatory reactions in the human body. Currently, the primary focus in this field is on biodegradable magnesium alloys. Mg^2^⁺ is the fourth most abundant cation in the human body, and magnesium is an essential metal element. Magnesium corrodes easily in various body fluids, and its Young’s modulus is similar to that of human bone, making it highly suitable as a degradable implant material. However, there are several major issues with magnesium alloys: (1) The degradation rate of magnesium alloys is too fast, leading to failure before the treatment is complete. (2) Excessive hydrogen gas generated during the degradation of magnesium alloys can form gas pockets under the skin, which can hinder the interaction between the implant and bone tissue, affecting bone healing. (3) The insufficient strength and toughness do not meet the required service life. Therefore, researchers are primarily focused on developing new types of biodegradable magnesium alloys that ensure sufficient strength and toughness, while reducing the degradation rate and minimizing hydrogen gas production. Compared to crystalline alloys, metallic glasses have a disordered microstructure without dislocations, grain boundaries, or other structural defects. As a result, magnesium-based metallic glass effectively prevents intergranular corrosion and slows down ion diffusion, demonstrating superior corrosion resistance compared to magnesium alloys, and reducing the issue of hydrogen gas pocket formation. Additionally, due to the absence of dislocations and grain boundaries, magnesium-based metallic glass also exhibits high strength.

Currently, biodegradable polymers and metals are materials with significant development potential in the field of biodegradable biomedical materials. Compared to biodegradable polymers, Mg-Zn-Ca alloys have more apparent advantages. They do not release acidic products, are easier to sterilize, and possess superior mechanical properties. Mg-Zn-Ca metallic glasses combine the advantages of traditional metals and polymers, boasting the excellent mechanical properties of metals along with their good biocompatibility and absorbability. Developing high-performance Mg-Zn-Ca metallic glasses is the future trend in the field of biodegradable materials. Over the past decade, various magnesium-based metallic glass systems have been developed, demonstrating significant potential applications in biodegradable orthopedic-fixation materials, cardiovascular stents, and other areas [57,58]. Different biological individuals and various usage sites within the same individual require different degradation absorption times for implants. To meet the application requirements in the biomedical field, developing new magnesium-based metallic glasses that do not contain toxic or harmful elements and have adjustable degradation rates is a crucial research direction. Studies have shown that preparing composite materials based on magnesium-based metallic glasses is an effective way to improve the overall performance of magnesium-based metallic glasses.

### 4.1. Development History of Magnesium-Based Metallic Glass

Research on magnesium-based metallic glasses began in the 1980s. In the initial research phase, the focus was on the fundamental physical properties of magnesium-based metallic glasses, such as glass-forming ability, resistivity, Hall coefficient, thermal power, and electron transport properties. Amorphous alloys exhibit stronger atomic bonding forces than crystalline alloys because amorphous alloys do not have dislocations, and therefore do not experience slippage caused by dislocation motion. As a result, the strength of metallic glasses in the same system is much higher than that of crystalline alloys. In 1991, A. Inoue [59] from Tohoku University in Japan first developed a 4 mm Mg-Cu-Y bulk magnesium-based metallic glass using copper mold casting (the T*g* of Mg-Cu-Y metallic glass is 427 K, and Tx is 448 K). This demonstrated that the Mg-Cu-Y metallic glass system has an excellent glass-forming ability and a large supercooled liquid region (ΔT = T*x* − T*g*). Subsequently, various bulk magnesium-based metallic glass systems were developed, including ternary systems like Mg-Ni-Nd [60], Mg-Ni-La [61], and Mg-Zn-Ca [27], as well as quaternary systems like Mg-Cu-Ni-Gd, Mg-Cu-Y-Zn [62], and Mg-Cu-Ag-Er. Studies have shown that adding transition elements like Ni and Cu, as well as rare earth elements, helps improve the glass-forming ability of magnesium-based metallic glasses, facilitating the preparation of large-sized metallic glasses. However, magnesium-based metallic glasses without toxic elements have weaker glass-forming abilities, a challenging issue when preparing non-toxic Mg-based metallic glasses in the field of biodegradable materials.

In 2005, Gu et al. [63] from the University of Virginia in the United States first prepared a 3 mm diameter Mg-Zn-Ca bulk metallic glass system using copper mold casting. In 1970, Mg-Zn amorphous ribbons were prepared using rapid solidification, but the glass-forming ability of Mg-Zn was weak, limiting the critical size. Studies have shown that adding an appropriate amount of Ca can affect the crystallization kinetics of Mg-Zn amorphous alloys, interfering with the intrinsic motion of Mg and Zn atoms, thereby increasing the crystallization time or reducing the crystallization rate and improving the material’s glass-forming ability (GFA). The results indicate that the strength of Mg-Zn-Ca metallic glass is 40% higher than that of crystalline magnesium alloys, and the system’s mass density is relatively small, making it a promising lightweight structural material with comprehensive properties.

The successful development of the non-toxic Mg-Zn-Ca metallic glass system has laid a crucial foundation for the development and application of magnesium-based metallic glasses in the biomedical field. From the perspective of biosafety, when selecting alloying elements for biomedical implant materials, priority should be given to beneficial nutritional elements while avoiding toxic or harmful metal elements. It is well known that zinc, as a vital trace element, is involved in the synthesis of proteins and various enzymes, and the activities of many enzymes (especially select enzymes in cartilage) are closely related to zinc [64,65]. Zinc plays an important regulatory role in human growth and development. Calcium, the most abundant mineral element in the human body, is an indispensable trace element essential for transmitting chemical signals within cells. Released calcium ions positively affect bone solidification. Therefore, the biosafety of calcium is guaranteed.

As a metallic glass system synthesized from elements entirely beneficial to the human body, the Mg-Zn-Ca metallic glass system has rapidly attracted attention and widespread interest in the biomedical field and is considered one of the most promising biodegradable biomedical materials [66].

### 4.2. Mechanical Properties of Magnesium-Based Metallic Glasses

Compared to crystalline alloys, Mg-based metallic glasses exhibit significantly improved mechanical properties. The changes in mechanical properties between the two alloys can be summarized as follows: (1) The tensile strength of Mg-based bulk metallic glasses is nearly three times that of crystalline Mg alloys. Due to their unique microstructure, metallic glasses lack initial structural defects such as grain boundaries and dislocations, resulting in a much higher strength than in crystalline alloys in the same system. (2) At the same tensile strength, Young’s modulus of Mg-based metallic glasses is almost three times that of crystalline magnesium alloys. Young’s modulus of Mg-based bulk metallic glasses is lower than that of Zr-based metallic glasses (as well as Ti-based, Fe-based, etc.). (3) Like crystalline alloys, there is a linear relationship between Young’s modulus and the tensile strength of metallic glasses.

### 4.3. Corrosion Resistance of Magnesium-Based Metallic Glasses

For implantable materials, controlling the degradation rate is crucial. Different implantation sites require different degradation rates. For instance, orthopedic implants need to maintain their integrity for the first three months after implantation to provide sufficient mechanical support and ensure tissue healing, with complete degradation within 12 months. Cardiovascular implants generally need to maintain their integrity for the first 18 months and complete degradation within 24 months [67]. Degradable materials are still in the early stages of development. There is no clear standard requirement for the degradation rate of biomedical materials. However, many scholars have proposed that for implantable materials, the degradation rate of biomedical degradable materials should be less than 0.2 mm/y [27]. Magnesium alloys, due to their excellent biocompatibility and degradability, are among the most promising degradable biomedical metals, particularly showing significant potential in orthopedic fixation and vascular stent applications. Currently, the main challenge hindering the use of magnesium alloys is their excessively rapid corrosion rate, which can lead to a premature loss of material mechanical properties, thereby failing to support tissue healing effectively. Magnesium in magnesium-based metallic glass reacts with water in the human body, producing hydroxides and hydrogen gas. Its rapid corrosion rate, accompanied by the generation of large amounts of gas, hinders tissue healing. Therefore, the mechanical properties, corrosion resistance, and biocompatibility of magnesium alloys are current research priorities. Compared to crystalline alloys, metallic glasses have a disordered microstructure without dislocations and grain boundaries, effectively preventing intergranular corrosion and slowing ion diffusion, thus exhibiting superior corrosion resistance [66]. Although Mg-based bulk metallic glasses have a higher corrosion resistance than crystalline alloys, the corrosion rate still does not meet clinical application requirements. Therefore, further improvement in the corrosion resistance of Mg-Zn-Ca metallic glasses is urgently needed.

Different elements have varying impacts on the corrosion behavior of Mg alloys. Adding alloying elements can reduce the corrosion tendency of the material by increasing its self-corrosion potential. Elements such as Y (−2.37 V), Nd (−2.43 V), and Ce (−2.48 V), which have electrochemical potentials similar to that of Mg (−2.37 V), are added to enhance corrosion resistance. Additionally, these alloying elements can significantly promote the increase in the quantity and variety of oxide films on the material’s surface. These oxide films act as a protective corrosion layer that adheres to the material surface, thereby providing some protection to the base material. The content of alloying elements also greatly affects the corrosion performance; excessive amounts can lead to the formation of a large amount of secondary phases, which can cause severe galvanic corrosion in the matrix, thus reducing the corrosion resistance of the material. Adding metals with high solubility in Mg, such as Sc (25.9% by mass), Gd (23.5% by mass), and Dy (25.3% by mass), can enhance corrosion resistance by reducing its internal galvanic corrosion in physiological environments.

### 4.4. Biocompatibility of Magnesium-Based Metallic Glasses

Bio-nutrient metallic elements (e.g., Ca, Sr, Zn, Mn) and non-toxic rare earth elements (e.g., Nd, Y, La), when added individually or together to the Mg matrix, not only do not cause harmful local tissue reactions but can also promote absorption by surrounding tissues. The addition of these bio-nutrient metallic elements and non-toxic rare earth elements improves the overall biocompatibility of the magnesium-based material. Small amounts of rare earth elements (REs) can induce grain refinement, solid solution strengthening, and second phase strengthening in magnesium-based metallic glasses, thereby improving their mechanical properties. Furthermore, low concentrations (0.2–1.0% by mass) of REs do not exhibit significant toxicity to cells; however, high concentrations (>10% by mass) may pose potential risks. The most commonly used REs include Nd, Sc, Y, Sm, Gd, and Dy.

**Figure 9:**
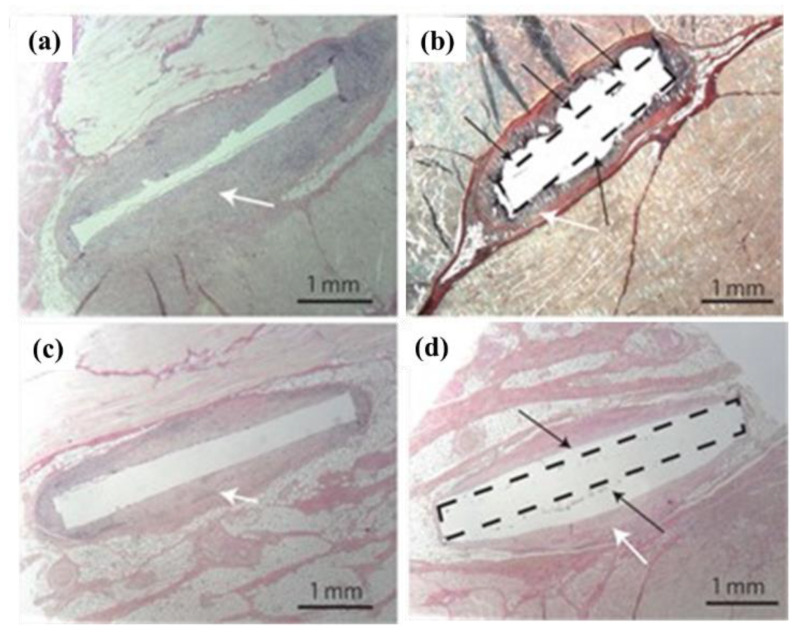
Animal studies of Mg-Zn-Ca metallic glass and Mg alloy reference sample [69]. Metallic glass (**a**) muscle after 27 days, (**b**) muscle after 27 days, (**c**) subcutis after 91 days with Mg alloy, (**d**) subcutis after 91 days.

As early as the 1930s, there was substantial research evidence indicating that magnesium (Mg) has good absorbability and compatibility as a fixation implant material [68]. Witte and colleagues discovered that after implanting Mg alloys, Mg^2^⁺ could effectively promote cartilage formation, aid in stimulating new bone formation, and accelerate fracture healing. In 2009, the team led by J.F. Loffler [69] first proposed the potential of Mg-Zn-Ca metallic glass as a degradable metal material for biomedical applications. They also pioneered the implantation of cast Mg-Zn-Ca metallic glass rods into animals to assess the biocompatibility and safety of Mg-Zn-Ca metallic glass. A comparative study was conducted by implanting Mg alloys (WZ21) and Mg-Zn-Ca metallic glass into pigs, observing tissue changes at 27 and 91 days post-implantation. The comparison revealed that Mg alloy implants caused noticeable foreign-body reactions and significant hydrogen gas cavities in the surrounding tissue. In contrast, Mg-Zn-Ca metallic glass implants did not exhibit hydrogen gas cavities or foreign-body reactions around them (Figure 9).

### 4.5. Future of Magnesium-Based Metallic Glasses

Currently, the main methods to improve the corrosion resistance of Mg-based bulk metallic glasses can be summarized as follows:

(1) Introducing Other Elements: Creating new metallic glass systems (Mg-Zn-Ca-M, where M represents other metals), such as Mg-Zn-Ca-Sr [70], Mg-Zn-Ca-Cu, Mg-Zn-Ca-Ga [71], and Mg-Zn-Ca-Ag [72]. The introduction of these elements can enhance the glass-forming ability of the Mg-Zn-Ca alloy system and promote the formation of a wider variety of oxides on the surface of Mg-Zn-Ca metallic glass, leading to the formation of a denser corrosion layer that protects the substrate [73].
(2) Developing Metallic Glass Composites: By introducing a second phase with better corrosion resistance, the self-corrosion potential of the composite can be increased and the corrosion resistance can be enhanced, thereby improving the overall corrosion resistance of the material [74,75].
(3) Surface Modification: Applying one or more thin films or coatings on the surface of the metallic glass to isolate it from corrosive media, thus protecting the material’s substrate and enhancing corrosion resistance [76,77]. Current surface-modification methods for Mg-Zn-Ca metallic glasses include chemical conversion, anodizing and micro-arc oxidation, plasma spraying, ion implantation, chemical deposition, electroplating, and composite coating [78,79,80]. These methods help maintain the intrinsic properties of the substrate material while effectively suppressing galvanic corrosion between the alloy and the liquid environment, regulating corrosion rates, and meeting other specific functional requirements.

## 5. Challenges in the Preparation Process

Introducing a porous structure into medical titanium alloys can offer several advantages:

(1) Compared to dense metals, metals with a three-dimensional interconnected porous structure provide a more favorable growth space for new bone tissue, which promotes better integration between the implant and bone tissue.
(2) Incorporating a three-dimensional interconnected porous structure into metals facilitates the transport of body fluids and nutrients, significantly accelerating tissue regeneration and shortening the healing period.
(3) Porosity is a critical parameter influencing the mechanical properties of metals; altering the porosity can further adjust the alloy’s mechanical properties, addressing the mismatch in mechanical performance between metal implants and human bone.

Introducing a porous structure into amorphous alloys requires maintaining the integrity of the amorphous phase. According to existing reports, the main methods currently used include melt foaming, infiltration casting, powder metallurgy with porogen addition, and additive manufacturing.

### 5.1. Melt Foaming Method

The in situ gas generation method and gas injection method share the same principle: the formation of pores originates from gases that cannot diffuse out during rapid cooling. During this rapid cooling process, gas bubbles merge randomly, resulting in a wide variation in pore sizes. The pore distribution is primarily influenced by the gas distribution within the melt, which is also highly disordered. This disordered and closed porous structure is not conducive to the practical application of amorphous alloys, leading to the gradual abandonment of this method.

### 5.2. Infiltration Casting Method

In the infiltration casting method, a porous material made of soluble particles is used as a template. High-temperature liquid metal is introduced into the template’s pores under pressure, and the soluble template is eventually dissolved to obtain a porous amorphous alloy. The process involves five steps:

(1) Loading soluble particles into a crucible for pre-pressing.
(2) Preforming the soluble particles through heating and holding.
(3) Injecting molten metal into the preformed template using pressure.
(4) Rapid cooling treatment.
(5) Dissolving the template to obtain the porous amorphous alloy.

Both the melt foaming and infiltration casting methods use metal melt as the research object, thus requiring a sufficiently high cooling rate during casting to prevent the formation of more thermodynamically stable crystalline phases when preparing porous metallic glasses. Therefore, the alloy system’s glass-forming ability significantly limits the final alloy’s achievable size. However, the powder sintering method largely overcomes the size limitations of amorphous alloys.

### 5.3. Powder Sintering with Porogen Addition

The space holder method, also known as the porogen method, involves mixing a porogen with metal powder and sintering the mixture. During or after sintering, the porogen is removed, leaving behind a porous material. By adjusting the quantity, size, and shape of the porogen, the material’s porosity, pore size, and pore shape can be controlled, further tuning the material’s mechanical properties.

In powder sintering, the powder is first pre-pressed into a shape, then sintered at a specific temperature and pressure. Adjusting sintering parameters and powder particle size controls the porosity and pore size of the porous material, thus altering its mechanical properties. During sintering, powder particles diffuse and bond, forming sintering necks that achieve metallurgical bonding; the pore-formation principle is based on the shrinkage between powder particles creating pores. This method is simple and efficient, but most of the resulting porous materials have a relatively low porosity, and the pore shapes are quite irregular.

In the fiber sintering method, metallic fibers are woven into a network and then sintered under certain pressure and temperature conditions. By controlling the diameter and length of the fibers, the porosity and mechanical properties can be adjusted [78]. This method requires the alloy to be first processed into metal fibers, which is more difficult and complex compared to powder processing.

Using powder metallurgy with porogen addition to prepare porous amorphous alloys allows for effective control over porosity and pore geometry, and it can overcome the size limitations of amorphous alloys, making it highly promising for application. During the preparation process, the following points should be noted:

(1) Ensure uniform mixing of the porogen and metal powder.
(2) Select a porogen with sufficient strength to prevent fragmentation during the sintering process.
(3) Avoid crystallization of the amorphous alloy during sintering and porogen removal while ensuring firm bonding of the amorphous alloy powder to maintain the strength of the porous alloy.
(4) Ensure a certain porosity to fully remove the porogen.

## 6. Summary and Outlook

Metallic glass features a short-range ordered, long-range disordered glassy structure without structural defects such as dislocations and grain boundaries, giving it the combined advantages of metals and glass, including high strength, toughness, and corrosion resistance. This novel alloy system, with its excellent comprehensive properties, has found applications in the field of biomedical materials. This paper reviews the applications of titanium-based bulk metallic glass (Ti-BMG) in weight-bearing long-term implants such as bone plates and screws, and magnesium-based metallic glass (Mg-BMG) in degradable implants, including bone screws, bone plates, and vascular stents, due to its degradability. However, as biomaterials, metallic glasses still face several challenges. Ti-BMG has a higher Young’s modulus than human bone, leading to a stress-shielding effect. Additionally, Ti-BMG’s biological inertness can cause aseptic loosening, and improving its antibacterial properties is another critical research direction. Mg-BMG’s rapid degradation rate leads to a premature loss of mechanical properties and excessive gas production, hindering tissue healing. Therefore, this paper proposes several key research directions for Ti-BMG and Mg-BMG:

(1) Introducing Porous Structures to Reduce Young’s Modulus: Introducing porous structures can lower Young’s modulus of metallic glass. However, this may degrade material properties, such as significantly reducing strength and corrosion resistance, which could compromise the long-term performance of titanium alloys. Thus, incorporating porous structures into Ti-BMG is an important research direction for reducing Young’s modulus.
(2) Enhancing Surface Bioactivity: To increase the bioactivity of implant surfaces, surface-modification methods such as ion implantation, electrochemical reactions, and hydrothermal treatments can be applied to Ti-BMG to enhance its surface bioactivity.
(3) Improving Antibacterial Properties of Medical Materials: Strategies include developing antibacterial coatings and incorporating antibacterial metal elements into the alloy to maintain the long-lasting antibacterial effects of the metallic biomaterial. Considering that some elements exhibit antibacterial properties only in specific forms, it is necessary to balance the antibacterial activity and mechanical properties of Ti-BMG.
(4) Modifying Mg-Based Metallic Glass: Mg-BMG as a medical implant material is still in its early stages. While it meets the strength requirements from a mechanical performance perspective, its corrosion resistance is still a concern. Therefore, further modifications to Mg-BMG are needed, such as enhancing its corrosion resistance through composite material development while maintaining biocompatibility and safety, allowing for controlled degradation rates and patterns.

## Figures and Tables

**Table 1:** Mechanical properties of some human bones [12].

		*E* (GPa)	*T* (MPa)	*C* (MPa)	*E* (%)
Shank	Femur	17.2	121.0	167.0	
	Shin bone	18.6	146.0	123.0	
Arm bone	Humerus	17.2	13.0	132.0	
	Radius	18.6	149.0	114.0	
	Ulna	18.0	148.0	117.0	
Vertebra	Jaw	0.23	3.1	10.0	
	Pelvic bone	0.16	3.7	5.0	
	Cancellous	0.09	1.2	1.9	
Cortical bone		7–30	50–150	100–230	1–3
Cancellous bone		0.1–0.5	10–20	2–12	5–7
Arthrodial cartilage		0.1	10–40		15–50
Dentin		18.2		295.0	

Note: *E* presents the modulus of elasticity, *T* presents the tensile strength, *C* presents the compressive strength, and *E* presents the elongation, respectively.
